# Inadequate Cancer Pain Control after Switching High-Dose Fentanyl to Hydromorphone Injection: A Case Series Highlighting Issues with Conventional Conversion Ratios

**DOI:** 10.1177/10966218251366061

**Published:** 2025-09-01

**Authors:** Naoki Suzuki, Atsuko Terasaki

**Affiliations:** ^1^ Department of Palliative Medicine, Yamagata Prefectural Central Hospital, Yamagata, Yamagata, Japan.; ^2^ Department of Pharmacy, Yamagata Prefectural Central Hospital, Yamagata, Yamagata, Japan.

**Keywords:** cancer pain, conversion ratios, fentanyl, hydromorphone, opioid rotation, opioid switching

## Abstract

Opioid conversion, particularly from high-dose intravenous (IV) fentanyl (>120 mg/day oral morphine-equivalent daily dose per referenced Japanese guidelines) to IV hydromorphone, presents clinical challenges due to inconsistent conversion ratios and lack of robust evidence. Specific approaches used in Japan may require careful evaluation. This report details two advanced cancer patients experiencing inadequate pain control after switching from high-dose IV fentanyl to IV hydromorphone. Conversions were based on calculations reflecting common Japanese practice. In both cases, pain worsened significantly, necessitating reversion to the original fentanyl regimen to regain acceptable analgesia. Conventional fentanyl-to-hydromorphone conversion ratios applied in Japan may underestimate the required hydromorphone dose when switching from high fentanyl baselines, risking therapeutic failure. These cases highlight the need for caution, consideration of potentially higher initial hydromorphone doses, close monitoring, and individualized strategies, including reverting to the prior opioid, for this specific rotation, especially in high-dose scenarios.

## Background 

Opioids are central to managing moderate-to-severe cancer pain, a common condition affecting a significant number of patients.^1^ However, effective pain management can be hindered by inadequate analgesia or intolerable side effects, often necessitating opioid switching—the practice of substituting one opioid for another or changing the administration route.^2^ While opioid switching is a widespread clinical strategy, determining appropriate conversion ratios between different opioids presents a considerable challenge, complicated by inconsistent practices among clinicians and across different countries.^3,4^ Despite the widespread use of conversion tables and guidelines, they often lack robust supporting evidence, and significant variability and uncertainty remain in clinical practice regarding opioid conversion.^4–8^

Conversion involving hydromorphone is particularly challenging, especially when switching from high-dose opioids. In this article, “high-dose” is defined as an oral morphine-equivalent daily dose (OMEDD) >120 mg/day, consistent with Japanese national guidelines referenced in studies.^9^ In such cases, consensus on appropriate conversion ratios remains lacking, and some reports suggest that doses significantly exceeding the calculated conversion amounts may be necessary when switching from high-dose opioids to hydromorphone, potentially requiring doses 2.5 times than predicted by standard conversion factors in certain cohorts.^10^ In particular, conversion ratios between fentanyl and hydromorphone show considerable variability. Although comparative studies have been conducted in noncancer pain settings, such as intensive care and pediatric populations, direct comparative research between fentanyl and hydromorphone in the context of cancer pain remains limited.^11,12^ Research suggests that established equianalgesic ratios may not accurately predict the required dose in complex clinical situations, such as in patients receiving high prior opioid doses or those with conditions such as cancer cachexia that can alter drug absorption and metabolism.^7,8,13–15^

This case series was prompted by clinical observations suggesting that conventional fentanyl-to-hydromorphone conversion ratios commonly used in Japan may result in inadequate pain control, particularly in high-dose settings.^16,17^ This case series presents two patients with advanced cancer pain who exhibited insufficient analgesia after switching from high-dose intravenous (IV) fentanyl to hydromorphone based on these conventional ratios. These cases illustrate the variability inherent in opioid conversion and highlight the limitations of standardized approaches, emphasizing the importance of clinical vigilance and individualized dose adjustments.

## Case Report

### Case 1

A 57-year-old woman presented with worsening abdominal pain and nausea due to malignant bowel obstruction secondary to pseudomyxoma peritonei, diagnosed six years prior. She had undergone tumor debulking surgery, experienced recurrence six months later requiring chemotherapy, and had multiple subsequent admissions for obstructive symptoms. Prior to this admission, her chronic pain had been managed on an outpatient basis with transdermal fentanyl. At this presentation, she had a small bowel stoma due to peritoneal dissemination, was nil per os (NPO) with nasogastric tube decompression, and exhibited marked cachexia with significant weight loss. Physical examination revealed upper abdominal firmness corresponding to palpable tumor lesions, with the patient reporting constant abdominal discomfort (numerical rating scale [NRS] 4–5/10) and intermittent severe cramping pain (NRS 7–8/10). Initial laboratory tests showed impaired renal function (creatinine 1.68 mg/dL; estimated glomerular filtration rate [eGFR] 26 mL/min/1.73 m^2^) with subsequent improvement to >30 mL/min within three days and preserved hepatic parameters (AST 24 U/L, ALT 43 U/L, total bilirubin 0.4 mg/dL). Albumin was 2.2 g/dL, reflecting severe cachexia. She was not taking any regular adjuvant analgesics, nor any medications known to significantly induce or inhibit CYP3A4.

**FIG. 1.**
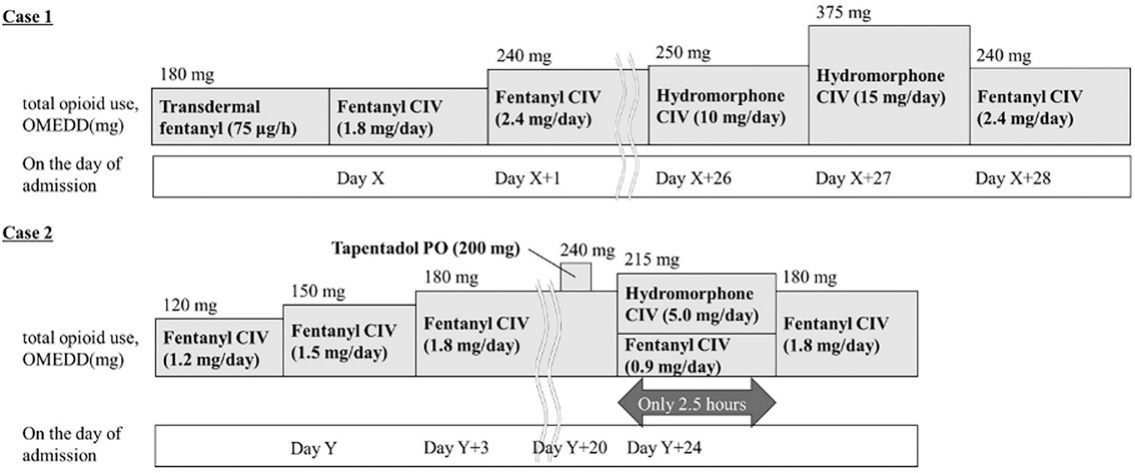
Clinical course of opioid administration. This figure illustrates the timeline of opioid dosages administered to the patients, including the switch from continuous intravenous (CIV) fentanyl to CIV hydromorphone and back to fentanyl. In this case series, opioid doses required for the conversion were determined using conversion tables and principles commonly utilized in Japanese clinical practice, as detailed in Table 1A.^16,17^ OMEDD, oral morphine equivalent daily dose; CIV, continuous intravenous infusion; PO, per os.

Upon admission, due to persistent symptoms and inability to use the transdermal route effectively, her opioid therapy was converted to a continuous IV fentanyl infusion, started at 1.8 mg/day (75 µg/h) and rapidly titrated to 2.4 mg/day (100 µg/h, OMEDD 240 mg) (Fig. 1). This regimen provided adequate pain control (NRS 2–3/10) for approximately four weeks. Subsequently, her pain worsened, particularly the intermittent cramping component likely related to bowel motility, alongside increasing discomfort from tumor progression. Due to this deterioration, perceived limitations of fentanyl for motility-related pain, the need for a higher concentration formulation (unavailable for fentanyl at high doses), and renal impairment precluding morphine, an opioid switch to IV hydromorphone was planned. No changes were made to other medications around the time of the switch. Based on conversion ratios commonly applied in Japan, approximating 0.6 mg IV fentanyl (25 µg/h) to 2.4 mg hydromorphone (Fentanyl:Hydromorphone ratio = 1:4) (detailed rationale and comparison with other ratios in Table 1),^16,17^ a direct switch from fentanyl 2.4 mg/day (100 µg/h) to hydromorphone 10 mg/day was performed (Fig. 1). However, this resulted in inadequate pain relief and frequent breakthrough pain. The dose was increased by 50% to 15 mg/day (Fig. 1), but the patient reported minimal benefit, stating the previous fentanyl regimen had provided better analgesia. Therefore, hydromorphone was discontinued the following day, and IV fentanyl 2.4 mg/day (100 µg/h) was restarted (Fig. 1), leading to satisfactory pain control similar to her prior experience.

**Table 1.** Conversion Ratios

A. Conversion ratios applied in the present cases.^16,17^			
	Equianalgesic doses (mg)		
Opioid	IV (mg/day)	PO (mg/day)	TD (µg/hr)
Morphine	15	30	N/A
Fentanyl	0.3	N/A	12.5
Hydromorphone	1.2	6	N/A
Tapentadol	N/A	100	N/A

B. Conversion ratios reported in the international literature.^10,18^			
	Equianalgesic doses (mg)		
Opioid	IV (mg/day)	PO (mg/day)	TD (μg/hr)
Morphine	10	30	N/A
Fentanyl	0.15	N/A	10
Hydromorphone	2	6	N/A
Tapentadol	N/A	100	N/A

IV, intravenous; PO, per os; TD, transdermal; N/A, not applicable.

### Case 2

A 61-year-old man with locally recurrent rectal cancer, initially treated with laparoscopic-assisted rectal resection and adjuvant chemoradiotherapy eight years prior, presented with significantly exacerbated chronic pain 12 days after undergoing ileal conduit creation for bilateral ureteral obstruction caused by a pelvic tumor mass. He exhibited marked cachexia with significant weight loss. His pain was primarily located in the right buttock and posterior thigh, consistent with likely tumor nerve infiltration based on imaging showing pelvic mass enlargement. Physical examination revealed slightly reduced superficial sensation in the affected area but no marked tenderness; vital signs were stable. Baseline laboratory results showed mild to moderate renal impairment and normal hepatic function (creatinine 1.22 mg/dL; eGFR 48 mL/min; AST 23 U/L; ALT 15 U/L; total bilirubin 0.5 mg/dL). Albumin was 2.8 g/dL. He described persistent pain fluctuating from NRS 4–5/10 to peaks of 7–8/10, with reported effectiveness of rescue opioids. He was not taking regular adjuvant analgesics (other than the recently added tapentadol) or medications known to significantly interact with CYP3A4.

Following palliative care consultation, his IV fentanyl infusion was titrated up from 1.2 mg/day (50 µg/h) to 1.8 mg/day (75 µg/h, OMEDD 180 mg) (Fig. 1). Despite this, pain control remained suboptimal. Recognizing a neuropathic component, oral tapentadol 200 mg/day (OMEDD 60 mg) was added but provided little benefit. Subsequently, the patient developed duodenal bleeding, which necessitated NPO status, leading to the discontinuation of all oral medications, including tapentadol. With pain control still inadequate on IV fentanyl 1.8 mg/day (75 µg/h) alone, and considering the need for high-dose capability and safety in potential renal instability, an opioid switch to IV hydromorphone was planned. No other changes were made to his analgesic regimen at the time of the switch. Based on common Japanese conversion principles (details and comparison with other conversion methods in Table 1),^16,17^ a gradual switch was initiated. With tapentadol already stopped due to the NPO requirement, fentanyl was reduced to 0.9 mg/day (37.5 µg/h), and hydromorphone 5 mg/day was started concurrently (Fig. 1). Within hours, however, his pain significantly worsened, and rescue medication became ineffective. The switch was therefore abandoned; hydromorphone was stopped, and IV fentanyl was restored to 1.8 mg/day (75 µg/h) (Fig. 1). This promptly improved pain control to the pre-switch baseline.

## Discussion

This case series highlights two significant clinical observations regarding opioid conversion for advanced cancer pain within Japan. First, switching from high-dose IV fentanyl to IV hydromorphone using conventional conversion ratios commonly applied locally may result in inadequate pain control. Second, these cases suggest that initiating hydromorphone at a dose higher than calculated by these standard ratios might be necessary for effective analgesia, particularly when converting from high prior opioid doses. These findings emphasize the known complexities and potential pitfalls of opioid rotation, placing the specific fentanyl-to-hydromorphone conversion in the Japanese context under scrutiny, given the background of recognized variability in equianalgesic tables and their supporting evidence.

The primary observation—the potential inadequacy of commonly referenced Japanese fentanyl-to-hydromorphone conversion ratios at high doses^16,17^ (defined in this context as >120 mg/day OMEDD based on referenced Japanese guidelines^9^)—is substantiated by the clear deterioration in pain control seen in both the present cases upon switching. This aligns with broader concerns about the limitations of standard conversion tables, which may not reliably apply to patients on chronic high-dose opioids.^7,8,13,14^ Direct comparative data for IV fentanyl to IV hydromorphone conversion in palliative care is scarce, often necessitating indirect calculations that may lack precision.^11,12,19^ Importantly, prospective studies involving opioid switching, particularly to hydromorphone from high baseline doses, have documented the frequent need for substantial dose increases beyond calculated equianalgesic amounts, with one study reporting required hydromorphone doses reaching 2.5 times those predicted by the utilized conversion factor.^10^ Notably, the conversion ratio applied in the present cases (approximately Fentanyl:Hydromorphone = 1:4), reflecting common local practice, appears significantly lower than ratios derived from some international data or indirect comparisons. While direct IV Fentanyl:Hydromorphone conversion data remain limited, extrapolations from IV hydromorphone to OMEDD ratios or direct comparisons in other contexts have suggested ratios considerably higher, such as 1:10 to 1:15 in certain reports or critical care settings, contrasting sharply with the 1:4 applied here.^11–13^ This discrepancy strongly suggests that reliance on these potentially underestimated ratios contributed to the observed treatment failure, warranting caution when applying them in high-dose fentanyl conversions in Japan.

This leads to the second key point: initiating hydromorphone at doses exceeding those derived from conventional Japanese ratios may be necessary when converting from high-dose fentanyl. This finding challenges standard guidelines that often advocate for a 25–50% dose reduction during opioid rotation to mitigate risks from incomplete cross-tolerance.^5^ Notably, the present cases demonstrated insufficient analgesia even without implementing this precautionary dose reduction. This experience mirrors reports where significant hydromorphone dose escalations were required post-switch, especially from high baseline opioid levels, sometimes needing doses substantially higher than initially calculated based on standard conversion factors.^7,10,14^ These observations suggest that for high-dose fentanyl-to-hydromorphone conversions, ratios significantly greater than the applied 1:4, perhaps approaching those used or suggested internationally (e.g., 1:10, 1:15), might have been more appropriate baseline estimates in these challenging scenarios.^11,12^ While incomplete cross-tolerance is a valid concern, these findings collectively imply that its impact may vary between specific opioid pairs and dose ranges, and that for high-dose fentanyl to hydromorphone conversions, the risk of underdosing due to potentially inaccurate baseline ratios might represent a more immediate clinical concern than the theoretical risk of toxicity from incomplete cross-tolerance if standard dose reduction practices are applied.

Several factors inherent to both fentanyl and hydromorphone likely contribute to the difficulties encountered in converting between these agents, particularly in the context of advanced cancer with marked cachexia, as observed in the present cases. Regarding fentanyl, its pharmacokinetics can be altered by cachexia. While cachexia may reduce CYP3A4 activity (the enzyme responsible for metabolizing fentanyl), it has also been paradoxically linked to delayed parenteral fentanyl clearance.^15,20^ Crucially, neither patient was taking medications known to significantly inhibit or induce CYP3A4. Furthermore, both patients exhibited marked hypoalbuminaemia (2.2 g/dL in Case 1 and 2.8 g/dL in Case 2, respectively). Given that fentanyl is approximately 80–85% protein-bound, whereas hydromorphone is less than 20% protein-bound, reduced protein binding due to hypoalbuminemia in these cachectic individuals could have disproportionately increased the free fraction of fentanyl.^21,22^ This elevation in unbound, pharmacologically active fentanyl might have contributed to its higher apparent efficacy and the perception that the fentanyl dose was providing more analgesia than its nominal dose would suggest. Therefore, this potential for altered fentanyl disposition due to both reduced clearance and increased free fraction in cachexia raises the possibility that the effective analgesic contribution of fentanyl was greater than predicted by the dose alone. Consequently, switching to hydromorphone based on standard conversion ratios, even those considered appropriate for noncachectic patients, might have resulted in a relative underdosing and the inadequate analgesia observed. For hydromorphone, significant challenges arise from the variability and uncertainty surrounding its equianalgesic ratios relative to other opioids.^23^ The combination of potentially altered fentanyl disposition in cachexia (both clearance and protein binding) and hydromorphone’s inherently problematic conversion ratios likely synergized to undermine the reliability of standard calculations in this specific rotation. Adding to these complexities, specifically in case 1, the patient experienced transient renal impairment with eGFR falling below 30 mL/min/1.73m^2^ at one point. This transient decline in renal function raises the possibility of hydromorphone-3-glucuronide (H3G) accumulation. H3G, a major metabolite of hydromorphone, is renally excreted and has been associated with neuroexcitatory phenomena, including paradoxical hyperalgesia, which can counteract the analgesic effects of the parent opioid.^24^ Although overt neurotoxicity such as myoclonus was not prominent, the potential for under-recognized early H3G-induced hyperalgesia contributing to the perceived analgesic failure with hydromorphone, even at increased doses, cannot be discounted. The combination of potentially altered fentanyl disposition in cachexia, hydromorphone’s inherently problematic conversion ratios, and the potential for H3G-mediated effects in the setting of renal impairment (as in Case 1), likely synergized to undermine the reliability of standard calculations in this specific rotation.

In addition to these pharmacokinetic and patient-specific factors, inter-individual variability in opioid response can also be influenced by pharmacogenetic differences. Polymorphisms in genes encoding opioid receptors, such as opioid receptor mu 1 (OPRM1), or those involved in drug metabolism and transport, have been reported to alter sensitivity and dose requirements for both fentanyl and hydromorphone.^25,26^ While specific genetic testing was not conducted in our cases, the potential for underlying pharmacogenomic variations contributing to the observed outcomes remains an important, albeit unquantified, consideration in complex opioid management and highlights an area for future research.

The clinical implications stemming from these observations and existing literature are significant, particularly for practice in Japan. This case series underscores the need for heightened vigilance and a potential reevaluation of standard conversion protocols when switching patients from high-dose IV fentanyl to IV hydromorphone. Based on the challenges observed in our cases, supported by literature indicating the frequent need for substantially higher hydromorphone doses than predicted by conventional ratios in palliative care, and noting that conversion practices in critical care settings have effectively utilized fentanyl-to-hydromorphone ratios that are considerably higher than those typically used in Japanese palliative practice, we propose that a pragmatic initial IV fentanyl-to-hydromorphone ratio of approximately 1:10 should be considered as a more appropriate starting point for patients on >120 mg/day OMEDD of fentanyl in the palliative setting, contrasting with the approximately 1:4 ratio applied in the present cases.^10–12^ This proposed ratio, while still requiring cautious application due to differing patient populations and contexts from the referenced critical care studies, aims to better align initial dosing with emerging evidence. However, initiating hydromorphone at such a higher ratio necessitates a strong emphasis on safety. Therefore, meticulous and frequent monitoring for adverse effects, particularly respiratory depression and signs of neurotoxicity such as myoclonus or excessive sedation, is paramount, especially during the initial 6–12 hours post-switch. Furthermore, to mitigate the risks of accidental over- or under-dosing, a staged conversion strategy (e.g., reducing the fentanyl dose by 50% while concurrently initiating hydromorphone at the calculated 1:10 proportion, followed by reassessment and further titration within four–six hours) could offer a safer approach, allowing for more individualized and cautious dose adjustments. Ultimately, the critical role of individual patient response, as highlighted by the successful reversion to fentanyl in our cases, emphasizes that hydromorphone might not be the optimal choice for every patient, reinforcing the importance of readiness to reassess and potentially reverse the switch if anticipated benefits are not realized or if safety concerns arise.

This report is limited by its nature as a case series involving only two patients, restricting the generalizability of findings. The interpretation, although based on prospectively documented courses, remains retrospective, and we cannot definitively exclude unmeasured confounding factors that might have influenced individual responses to opioid switching. Furthermore, these observations stem from a specific Japanese clinical context. Despite these limitations, the cases provide valuable clinical insights. Switching from high-dose IV fentanyl to hydromorphone in patients with advanced cancer pain poses significant challenges, and conversion ratios commonly applied in Japan may lead to underdosing and inadequate analgesia. Clinicians should exercise caution with these specific conversions, consider patient-specific factors (high prior dose, cachexia) potentially necessitating higher initial hydromorphone doses, monitor closely, and maintain flexibility to adjust or revert therapy based on individual response. Further research is needed to establish more reliable, context-specific conversion guidelines for this opioid rotation.

## Research Ethics and Patient Consent

Our institution does not require ethical approval for reporting individual cases or case series. The patients and their families have given written informed consent for publication.

## Abbreviations Used

CIV: continuous intravenous
eGFR: estimated glomerular filtration rate
H3G: hydromorphone-3-glucuronide
IV: intravenous
NPO: nil per os
NRS: numerical rating scale
OMEDD: oral morphine-equivalent daily dose
OPRM1: opioid receptor mu 1
PO: per os

## References

[1] Portenoy RK. Treatment of cancer pain. Lancet 2011;377(9784):2236–2247. 21704873 10.1016/S0140-6736(11)60236-5

[2] Mercadante S, Bruera E. Opioid switching in cancer pain: From the beginning to nowadays. Crit Rev Oncol Hematol 2016;99:241–248. 26806145 10.1016/j.critrevonc.2015.12.011

[3] Reddy A, Sinclair C, Crawford GB, et al. Opioid rotation and conversion ratios used by palliative care professionals: An international survey. J Palliat Med 2022;25(10):1557–1562. 35930252 10.1089/jpm.2022.0266 PMC9836667

[4] Davis MP, Davies A, McPherson ML, et al. Opioid analgesic dose and route conversion ratio studies: A scoping review to inform an eDelphi guideline. Support Care Cancer 2024;32(8):542. 39046534 10.1007/s00520-024-08710-0

[5] Davis MP, Davies A, McPherson ML, et al. Opioid conversion in adults with cancer: MASCC-ASCO-AAHPM-HPNA-NICSO guideline. Support Care Cancer 2025;33(3):243. 40029420 10.1007/s00520-025-09286-z

[6] Mercadante S, Caraceni A. Conversion ratios for opioid switching in the treatment of cancer pain: A systematic review. Palliat Med 2011;25(5):504–515. 21708857 10.1177/0269216311406577

[7] Schuster M, Bayer O, Heid F, et al. Opioid rotation in cancer pain treatment. Dtsch Arztebl Int 2018;115(9):135–142. 29563006 10.3238/arztebl.2018.0135 PMC5876542

[8] Knotkova H, Fine PG, Portenoy RK. Opioid rotation: The science and the limitations of the equianalgesic dose table. J Pain Symptom Manage 2009;38(3):426–439. 19735903 10.1016/j.jpainsymman.2009.06.001

[9] Sumimoto H, Hayashi K, Kimura Y, et al. Factors associated with cancer-related pain requiring high-dose opioid use in palliative cancer patients. Palliat Med Rep 2021;2(1):237–241. 34927147 10.1089/pmr.2021.0037 PMC8675226

[10] Wong AK, Klepstad P, Rubio JP, et al. Opioid switch dosing in chronic cancer pain: A prospective longitudinal study. J Palliat Med 2024;27(3):388–393. 37955655 10.1089/jpm.2023.0541

[11] Kovacevic MP, Szumita PM, Dube KM, et al. Transition from continuous infusion fentanyl to hydromorphone in critically ill patients. J Pharm Pract 2020;33(2):129–135. 29996718 10.1177/0897190018786832

[12] Harkin M, Miller JL, Neely SB, et al. Conversion from continuous infusion fentanyl to continuous infusion hydromorphone in the pediatric intensive care unit. Ann Pharmacother 2021;55(12):1439–1446. 33745290 10.1177/10600280211003170

[13] Reddy A, Vidal M, Stephen S, et al. The conversion ratio from intravenous hydromorphone to oral opioids in cancer patients. J Pain Symptom Manage 2017;54(3):280–288. 28711751 10.1016/j.jpainsymman.2017.07.001 PMC13267606

[14] Weinstein SM, Shi M, Buckley BJ, et al. Multicenter, open-label, prospective evaluation of the conversion from previous opioid analgesics to extended-release hydromorphone hydrochloride administered every 24 hours to patients with persistent moderate to severe pain. Clin Ther 2006;28(1):86–98. 16490582 10.1016/j.clinthera.2006.01.010

[15] Suno M, Endo Y, Nishie H, et al. Refractory cachexia is associated with increased plasma concentrations of fentanyl in cancer patients. Ther Clin Risk Manag 2015;11:751–757. 26056457 10.2147/TCRM.S79374 PMC4431473

[16] Hiratsuka Y, Tagami K, Inoue A, et al. Prevalence of opioid-induced adverse events across opioids commonly used for analgesic treatment in Japan: A multicenter prospective longitudinal study. Support Care Cancer 2023;31(12):632. 37843639 10.1007/s00520-023-08099-2 PMC10579154

[17] Tagami K, Chiu SW, Kosugi K, et al. Cancer pain management in patients receiving inpatient specialized palliative care services. J Pain Symptom Manage 2024;67(1):27–38.e1. 37730073 10.1016/j.jpainsymman.2023.09.015

[18] Australian and New Zealand College of Anaesthetists. Opioid dose equivalence calculation table. 2021. Available from: https://www.anzca.edu.au/getContentAsset/fbd6254a-05be-48eb-a50f-a6e85d89d4db/80feb437-d24d-46b8-a858-4a2a28b9b970/PS01(PM)-(Appendix)_Opioid-Dose-Equivalence-Calculation-Table.PDF [Last accessed: April 28, 2025].

[19] Dinges HC, Schubert AK, Rücker G, et al. Equianalgesic potency ratios of opioids used in patient-controlled analgesia: A network meta-analysis. J Opioid Manag 2022;18(6):567–586. 36523208 10.5055/jom.2022.0751

[20] Davis M. Fentanyl pharmacokinetic paradoxical in cancer cachexia. J Pain Symptom Manage 2024;68(1):e78. 38570173 10.1016/j.jpainsymman.2024.03.019

[21] Meuldermans WE, Hurkmans RM, Heykants JJ. Plasma protein binding and distribution of fentanyl, sufentanil, alfentanil and lofentanil in blood. Arch Int Pharmacodyn Ther 1982;257(1):4–19. 6214227

[22] Sarhill N, Walsh D, Nelson KA. Hydromorphone: Pharmacology and clinical applications in cancer patients. Support Care Cancer 2001;9(2):84–96. 11305075 10.1007/s005200000183

[23] Murray A, Hagen NA. Hydromorphone. J Pain Symptom Manage 2005;29(Suppl 5):S57–S66. 15907647 10.1016/j.jpainsymman.2005.01.007

[24] Nakatani T, Shiosakai K, Hashimoto T, et al. Steady-state pharmacokinetics of intravenous hydromorphone in Japanese patients with renal impairment and cancer pain. J Palliat Med 2023;26(6):768–775. 36579915 10.1089/jpm.2022.0289 PMC10278029

[25] Takemura M, Niki K, Okamoto Y, et al. Comparison of the effects of OPRM1 A118G polymorphism using different opioids: A prospective study. J Pain Symptom Manage 2024;67(1):39–49.e5. 37757956 10.1016/j.jpainsymman.2023.09.017

[26] Takashina Y, Naito T, Mino Y, et al. Impact of CYP3A5 and ABCB1 gene polymorphisms on fentanyl pharmacokinetics and clinical responses in cancer patients undergoing conversion to a transdermal system. Drug Metab Pharmacokinet 2012;27(4):414–421. 22277678 10.2133/dmpk.dmpk-11-rg-134

